# Development of a scale to evaluate negative menstrual attitudes among Nepalese women

**DOI:** 10.1186/s12978-022-01426-6

**Published:** 2022-05-16

**Authors:** Rina Kawata, Masayuki Endo, Shiba Kumar Rai, Kazutomo Ohashi

**Affiliations:** 1 Division of Health Science, Graduate School of Medicine, Osaka University, 1-7, Suita, Osaka, 565-0871 Japan; 2 Nepal Medical College, Attarkhel, Gokarneswor Municipality-8, Kathmandu, Nepal; 3 Faculty of Global Nursing, Otemae University, 2-1-88, Cyuoku Otemae, Osaka, 540-0008 Japan

**Keywords:** Negative menstrual attitudes, Nepalese women, Traditional menstrual practices, Menstrual pain, Reproductive health

## Abstract

**Background:**

Menstrual attitudes include negative and positive perceptions of menstruation and are associated with reproductive health, underscoring the importance of its evaluation in women. Given that social and cultural factors affect menstrual attitudes, developing evaluation methods specific to distinct societal cultures is necessary.

**Methods:**

We developed a scale based on the menstrual attitude questionnaire, which is the most widely used measure of menstrual attitudes, to evaluate negative menstrual attitudes among Nepalese women in consideration of Nepalese cultural backgrounds and literacy. In total, 352 Nepalese women aged 20–45 years living in urban or suburban areas in Nepal were enrolled in the study. Participants were randomly divided into two groups using the permuted block method. Using the prototype, Group 1 (n = 176) was evaluated with an exploratory factor analysis to develop a reliable scale, and Group 2 (n = 176) was evaluated with a confirmatory factor analysis to confirm the structure of negative menstrual attitudes. Further, we preliminarily examined the relationship of negative menstrual attitudes with the frequency of traditional menstrual practices and intensity of menstrual pain, which are significant reproductive health issues in Nepal, in Group 2.

**Results:**

We developed a 15-item, 3-factor scale to evaluate negative menstrual attitudes among Nepalese women using exploratory factor analysis. The first, second, and third factors were “Natural event” (α = 0.82), “Debilitating event” (α = 0.76), and “Bothersome event” (α = 0.69), respectively. Confirmatory factor analysis revealed that negative menstrual attitudes comprised a 3-factor structure. Participants who performed three traditional menstrual practices (“stay in their own house”, “go to public spaces”, and “contact with others generally”) demonstrated significantly higher scores for negative menstrual attitudes than did non-performers. Negative menstrual attitudes were positively correlated with the intensity of menstrual pain (r = 0.558).

**Conclusions:**

This study is the first to evaluate menstrual attitudes and analyse its factor structure among Nepalese women. In the future, the relationship between accurately evaluated negative menstrual attitudes and reproductive health issues should be examined more comprehensively.

## Plain Language Summary

For sexually mature women, menstruation may be considered burdensome, but its absence may also be a source of concern. Perceptions regarding menstruation are collectively referred to as menstrual attitudes. Previous studies have reported that negative menstrual attitudes are associated with various reproductive health issues in women and vary according to social and cultural backgrounds. To adequately evaluate negative menstrual attitudes among Nepalese women (20–45 years old), we developed a 15-item scale that considers their cultural background and literacy. We observed that attitudes toward menstruation among Nepalese women consisted of three emotions: one positive emotion, including “menstruation is a natural phenomenon”, and two negative emotions, including “menstruation is debilitating” and “menstruation is bothersome”. In several developing countries, menstruating women are considered unclean, and various restrictions are imposed on women during menstruation (traditional menstrual practices). Nepalese women with strong negative menstrual attitudes, as assessed by our scale, engaged in several traditional menstrual practices with higher frequency. We also observed that women with more negative menstrual attitudes presented with more severe menstrual pain. Our scale was developed to be compatible with the values and literacy of Nepalese women and was easy to answer. We believe that reducing negative emotions toward menstruation will contribute to reducing the frequency of traditional menstrual practices and menstrual pain, thereby facilitating school attendance and entry into the workforce among women in Nepal.

## Background

Although menstruation is a natural physiological phenomenon, menstruating women are considered religiously and socially “unclean” in several cultures, leading to limitations in activities of daily living [1]. Such cultural and social attitudes toward menstruation adversely affect women’s attitudes and beliefs toward menstruation as well [2]. Menstrual attitudes include both negative (menstruation is debilitating, bothersome, and stressful) and positive (menstruation is a natural event) attitudes [3]. Negative menstrual attitudes have been associated with the severity of menstruation-related symptoms, including premenstrual symptoms and menstrual pain, [4, 5]. Conversely, positive menstrual attitudes have been associated with scientifically evidenced menstrual coping strategies [6]. Therefore, evaluating menstrual attitudes across different cultures is key to ensure that women of reproductive age lead healthy lives both physically and mentally during menstruation.

Brooks-Gunn and Ruble developed the Menstrual Attitudes Questionnaire (MAQ) to evaluate menstrual attitudes [3], which has been translated into several languages and is used in several countries, including India [7], Jordan [6], Taiwan [8], Japan [9], Turkey [2], Germany [10], and Greece [11]. Although other assessment methods, such as questions about menstruation developed by the World Health Organization [1], the Adolescent Menstrual Attitude Questionnaire (AMAQ) for pre- and post-menstrual early adolescents [12], and questions about images of negative menstruation [13], have been reported, the MAQ remains the most widely used international standard measure for comparing menstrual attitudes among nations due to its validity and reliability [7]. However, as menstrual attitudes differ between cultures [5], it is essential to use a scale that reflects the cultural background of the relevant society when evaluating menstrual attitudes.

Particularly, the behaviour of Nepalese women during menstruation is restricted both religiously and socially. Traditional menstrual practices, which impede access to sanitary water [14] and lack hygienic menstrual coping strategies, contribute to an increase in health issues, including dysmenorrhoea [15] and urinary tract infections [16]. In addition, 71.5% of Nepalese adolescent girls complained of menstrual pain, which has been associated with absenteeism from school, thereby becoming a social problem [17]. Crawford et al. reported that such traditional menstrual practices cause negative menstrual attitudes, such as fear, stress, inconvenience, and embarrassment, among Nepalese women from menarche [18]. However, only a part of the MAQ has been used to assess menstrual attitudes, resulting in an insufficient examination.

Accordingly, there is an urgent need to address reproductive health issues and develop interventions to improve menstrual attitudes among Nepalese women. Therefore, this study aimed to develop a scale to evaluate the menstrual attitudes of Nepalese women and analyse the structure of their menstrual attitudes. To examine the usefulness of the scale, we preliminarily examined the relationship of scale scores with the frequency of traditional menstruation practices and severity of menstrual pain, which are reported to affect menstrual attitudes.

## Methods

### Scale development

Researchers of Osaka University first translated the 35-item MAQ with a 5-factor structure developed by Brooks-Gunn to Japanese (1st edition protocol). The first author and two Nepalese research collaborators, who lived in Japan and understood Japanese well, then examined whether the questions of the 1st edition protocol could be applied to menstrual attitudes in Nepal and created the 2nd edition protocol in Japanese, consisting of a 20-item questionnaire with a 3-factor structure. In addition, we removed items from the 2nd edition protocol that may have been difficult for Nepalese women to understand and unified the expressions of the subjects and predicates. Afterwards, the contents were reviewed in consideration of the Nepalese culture, and an easier expression was implemented due to low literacy among Nepalese women. Subsequently, we created the 3rd edition protocol in Japanese, in which the response format was changed from a 7-point scale on the MAQ to a 5-point scale that comprised 17 questions, allowing Nepalese women to easily answer the questionnaire. Finally, the 3rd edition protocol was translated from Japanese to Nepali by the first author and two Nepalese collaborators (3rd edition protocol in Nepalese). To examine the comprehensibility of the 3rd edition protocol, the first preliminary survey was conducted on 108 Nepalese women living in Japan, and a second preliminary survey was conducted on eight Nepalese women living in Nepal. In this preliminary survey, the researchers and participants exchanged opinions on the perspective of cultural values and literacy in Nepalese women and carefully examined the contents of the question items for the creation of the 4th edition protocol in Nepalese. The 4th edition protocol comprised three subscales (debilitating event, bothersome event, and natural event) and 17 items as shown in Table 1. The debilitating event consists of seven items that deem menstruation primarily physically debilitating. Women feel more tired, unhealthier, greater changes in physical condition, and more behavior restriction than usual during menstruation. The bothersome event consists of five items that deem menstruation primarily psychologically bothersome. Women believe that menstruation is something to endure, a disadvantage for women, and a sign of non-pregnancy. The natural event consists of five items that deem menstruation primarily a physically and psychologically natural event. Women believe that menstruation is a sign of good health and womanhood. Scores for the natural event were treated as inverted items, such that the total score indicated negative menstrual attitudes. Responses were scored using a 5-point Likert scale (1 = strongly disagree, 2 = disagree, 3 = neutral, 4 = agree, and 5 = strongly agree).

**Table 1:** Subcategories and items of the 4th edition protocol and its item analysis (n = 176, Group 1)

Items	M (SD)	Floor	Celling
		M + 1SD	M − 1SD
Subcategory 1 debilitating event (7 items)			
1. I think more tired than usual during menstruation	3.1 (1.2)	1.9	4.3
2. I think I expect extra consideration from people around me during menstruation	3.4 (1.2)	2.3	4.6
3. I feel that changes in physical condition during menstruation are greater than changes in normal physical condition	3.3 (1.1)	2.2	4.4
4. I think unhealthier than usual during menstruation	3.1 (1.1)	2.0	4.2
5. I think I can’t do as usual behaviors during menstruation	2.6 (1.1)	1.5	3.8
6. I think it is better to avoid certain behaviors during menstruation	2.9 (1.2)	1.8	4.2
7. I easy not to feel well than usual before menstruation	2.7 (1.1)	1.6	3.8
Subcategory 2 Bothersome event (5 items)			
8. I think menstruation is something I just have to put up with	3.8 (0.9)	3.0	4.7
9. I can’t feel good during menstruation	2.8 (1.2)	1.7	4.1
10. I think men have advantage in not having menstruation	3.4 (1.2)	2.3	4.7
11. I think I hope it will be possible to get a short menstrual period	3.1 (1.3)	1.8	4.4
12. I think the only thing menstruation is good for is to let me know I’m not pregnant	3.7 (1.0)	2.7	4.6
Subcategory 3 natural event (5 items, reversed item)			
13. I think menstruation is realized womanhood	2.0 (0.8)	1.3	2.7
14. I think I am more strongly aware of my health condition than usual by menstruation	2.1 (0.9)	1.2	2.8
15. I think menstruation is a way to inform me of my health condition	1.9 (0.7)	1.3	2.5
16. I think menstruation is phenomenon which keep all of life	2.1 (0.9)	1.3	2.9
17. I think menstruation is a sign of good health	1.8 (0.7)	1.3	2.4

*M* mean, *SD* standard deviation

### Procedure

This cross-sectional study was conducted between August 2018 and December 2018. We recruited 355 Nepalese women aged 20–45 years living in Pokhara of the Kaski District (metropolitan area) and Damauli of the Tanahu District (municipal area) [19]. Pokhara is the second largest city in Nepal located 200 km west of the capital, Kathmandu, and has a total population of 402,995 [20]. Meanwhile, Damauli (Vyas) is in a municipal area adjacent to the eastern part of the Kaski District and has a total population of 70,335 [20].

A research collaborator requested Nepalese women to participate in the research. To recruit Nepalese participants, two Nepalese coordinators of the Japan International Cooperation Agency (JICA) helped with the study. We collected the participants via snowball sampling, and recruitment was done in community meetings, such as the Lions club in Pokhara and mother-group meetings in Damauli. We also used flyers and posters that include the contact addresses and telephone numbers of the research collaborators whom volunteers could contact.

Following recruitment, the participants gathered individually at a meeting place in their respective residential area. All participants received a face-to-face explanation regarding the purposes and methods of the study before providing their written consent. Questionnaires were then distributed to women who consented to participate in the study. The research collaborator read out the questionnaire, and the participants filled in their responses. When participants could not sufficiently understand the contents of the questionnaire, the research collaborator provided an additional explanation. For illiterate participants, the research collaborator wrote the responses on their behalf. To reduce response bias, the research collaborators and researchers discussed the assistance method in advance and tried to respect the participants’ will. After the participants had completed the questionnaire, the research collaborator placed the collection box and left the survey site, and the participants posted their questionnaires in the collection box. The research collaborator collected the questionnaires from the collection box afterwards. We considered the recovery of the questionnaire as consent to participate in the research.

### Questionnaire

The questionnaire consisted of (1) demographic characteristics (age, religion, educational level, castes, and number of children), (2) negative menstrual attitudes (4th edition protocol), (3) frequency of traditional menstrual practices, and (4) intensity of menstrual pain.

Based on the previous studies of traditional menstrual practices in Nepal [14, 18, 21, 22], we extracted two categories of restrictions that negatively affected women’s emotions. The first category was living place restrictions, defined as not staying in their own house, not going to public spaces, not staying in others’ houses, or not using a bedroom or kitchen. The second category was restrictions on contact with others, defined as no general contact with others, no contact with the male family, or not attending to the male family. Moreover, we examined the restrictions on religious behaviours, which were reported to be less strongly associated with negative emotions. Restrictions on religious behaviours were defined as not going to temples, not joining religious events, or not attending a wedding ceremony. Participants were questioned about the frequency of 11 menstrual traditional practices, including five items of living place restrictions, three items of restrictions on contact with others, and three items of restrictions on religious behaviours. Responses regarding frequency were scored on a 5-point scale (1 = never, 2 = rarely, 3 = sometimes, 4 = often, and 5 = always). A higher score indicated a higher frequency of traditional menstrual practices. Responses on the intensity of menstrual pain were similarly scored on a 5-point scale (1 = none, 2 = mild, 3 = moderate, 4 = severe, and 5 = very severe).

### Analysis

Of the 355 participants, only 352 complete responses were included in the analysis. An exploratory factor analysis with generalised least squares and promax rotation was first performed to develop a menstrual attitude scale based on the 4th edition protocol. The sample size was determined to be 170, which was 10 times the number of question items (17 items) in the 4th edition protocol, following the 10 times rule sample size [23]. We then randomly divided the 352 participants into two groups using the permuted block method, which was a replacement block method with a block size of 2 (Table 2). First, we divided the 252 participants in the Pokhara group and 100 participants in the Damauli group in numerical order. A was defined as the Group 1 and B as the Group 2. Specifically, half of the random numbers in each group were generated and assigned to each group in numerical order, with AB above 0.5 and BA below 0.5. The group assigned to A was Group 1, and the group assigned to B was Group 2. In total, data from 176 participants in Group 1 were used in exploratory factor analysis. Floor and ceiling effects were also confirmed using the scores from the 4th edition protocol in Group 1 (n = 176) (Table 1). After promax rotation, we selected the items with factor loadings > 0.35, > 0.40, or 0.45, and the number of factors was determined using a scree plot. Furthermore, reliability was examined using Cronbach’s α.

**Table 2:** Background characteristics of women with complete responses

	Group 1 (n = 176)	Group 2 (n = 176)	Total (n = 352)
Age (years)	33.4 ± 8.3	32.8 ± 8.0	33.12 ± 8.2
Residence n (%)			
Pokhara	126 (71.6)	126 (71.6)	252 (71.6)
Damauli	50 (28.4)	50 (28.4)	100 (28.4)
Religions n (%)			
Hindus	161 (90.3)	160 (90.9)	321 (91.2)
Buddhist	11 (6.3)	7 (4.0)	18 (5.1)
Muslim	0 (0.0)	1 (0.6)	1 (0.3)
Christian	2 (1.1)	5 (2.8)	7 (2.0)
Other	2 (1.1)	3 (1.7)	5 (1.4)
Education level n (%)			
Low	89 (50.6)	90 (51.1)	179 (50.8)
Non-educated	23 (13.1)	20 (11.4)	43 (12.2)
Primary	15 (8.5)	21 (11.9)	36 (10.2)
Secondary	51 (29.0)	49 (27.8)	100 (28.4)
High	87 (49.4)	86 (48.9)	173 (49.2)
High school	36 (20.5)	31 (17.6)	67 (19.0)
Nursing school	4 (2.3)	1 (0.6)	5 (1.4)
Vocational degree	3 (1.7)	4 (2.3)	7 (2.0)
Bachelors	35 (19.9)	33 (18.8)	68 (19.3)
Masters	9 (5.1)	17 (9.7)	26 (7.4)
Castes n (%)			
Brahmin and Chhetri	63 (35.8)	72 (40.9)	135 (38.4)
Adibashi-Janajati	100 (56.8)	88 (50.0)	188 (53.4)
Dalit	13 (7.4)	16 (9.1)	29 (8.2)
Intensity of menstrual pain n (%)			
None	47 (26.7)	43 (24.4)	90 (25.6)
Mild	32 (18.2)	28 (15.9)	60 (17.0)
Moderate	39 (22.2)	42 (23.9)	81 (23.0)
Severe	40 (22.7)	37 (21.0)	77 (21.9)
Very severe	18 (10.2)	26 (14.8)	44 (12.5)

To examine the factorial structure of negative menstrual attitudes among Nepalese women, a confirmatory factor analysis was conducted using the scores in Group 2 (n = 176). In the confirmatory factor analysis, “Q17, 16, 15, 14, and 13” were assumed to be strongly influenced by the “natural event” factor; “Q5, 4, 9, 6, and 1” by the “debilitating event” factor; and “Q10, 2, 3, 8, and 11” by the “bothersome event” factor. We subsequently drew a path diagram with two headed arrows pointing from the factor to each observed variable, assuming that the three factors were correlated. The estimates of these three factors were obtained by generalised least squares. The goodness-of-fit of the model was examined using the chi-square value (CMIN), Akaike’s information criterion (AIC), goodness-of-fit index (GFI), adjusted GFI (AGFI), comparative fit index (CFI), and root mean square error of approximation (RMSEA).

In addition, we analysed the structure of negative menstrual attitudes among Nepalese women; however, the criterion validity may be insufficient due to the absence of a gold standard. Therefore, we preliminarily examined the relationship between negative menstrual attitudes evaluated using the 15-item scale and two parameters—traditional menstrual practices and menstrual pain—which are related to reproductive health. We also examined the relationship between negative menstrual attitudes and frequency of traditional menstruation practices, which result in reproductive health issues among Nepalese women. Answers regarding the frequency of traditional menstrual practices were divided into two groups: those in non-performers (answer 1) and those in performers (answers 2 to 5). Scores of the 15 items in the 4th edition of the protocol, which was abstracted in the exploratory factor analysis, were calculated to examine the score differences between performers and non-performers of each traditional menstrual practice. The scores were also compared between non-performers and performers in Group 2 using the Mann–Whitney U test, and the relationship between the total scores of the 15 items and intensity of menstrual pain was examined using Spearman's correlation coefficient. Statistical analysis was conducted using the SPSS ver. 27 and SPSS AMOS ver. 27 (IBM, Tokyo, Japan) software, and statistical significance was set at 5%.

## Results

The background characteristics of Group 1 are presented in the left column of Table 1. Table 2 shows the means ± standard deviation (SD) of the scores in the 4th edition protocol. There were no items in the 4th edition protocol with a floor or ceiling effect, when the ceiling effect was determined as exceeding 5 of the mean + 1SD and the floor effect as less than 1 of the mean − 1SD. Exploratory factor analysis with promax rotation was performed using 17 items (Table 3). Factorability was confirmed using the Kaiser–Meyer–Olkin (KMO) measure of sampling adequacy (0.865) and Bartlett’s test of sphericity (P < 0.001). The scree plot indicated the following three factors, and the number of factors to be extracted was set to 3 using the Kaiser–Guttman criterion. Accordingly, we adopted 15 items with factor loadings > 0.40. Based on the exploratory factor analysis, the scale of negative menstrual attitudes among Nepalese women consisted of three subcategories with 15 items. The first, second, and third subcategories were termed ‘Natural event’ (α = 0.82), ‘Debilitating event’ (α = 0.76), and ‘Bothersome event’ (α = 0.69), respectively. This resulted in the development of a 15-item scale to assess negative menstrual attitudes among Nepalese women.

**Table 3:** Exploratory factor analysis of the 4th edition protocol (n = 176, Group 1)

Item	M (SD)	Factor		
		I	II	III
**Factor 1 Natural event (5 items, reversed item) α = 0.82**				
17. I think menstruation is a sign of good health	2.0 (0.6)	**0.848**	0.008	− 0.058
16. I think menstruation is phenomenon which keep all of life	2.0 (0.8)	**0.799**	− 0.029	− 0.137
15. I think menstruation is a way to inform me of my health condition	2.0 (0.6)	**0.722**	0.047	− 0.055
14. I think I am more strongly aware of my health condition than usual by menstruation	2.0 (0.8)	**0.570**	− 0.017	− 0.370
13. I think menstruation is realized womanhood	2.0 (0.7)	**0.496**	0.063	− 0.179
**Factor 2 Debilitating event (5 items) α = 0.76**				
5. I think I can’t do as usual behaviors during menstruation	2.0 (1.2)	0.188	**0.756**	− 0.321
4. I think unhealthier than usual during menstruation	3.0 (1.1)	0.033	**0.709**	0.216
9. I can’t feel good during menstruation	2.0 (1.2)	− 0.219	**0.569**	0.343
6. I think it is better to avoid certain behaviors during menstruation	3.0 (1.2)	− 0.014	**0.558**	0.022
1. I think more tired than usual during menstruation	3.0 (1.2)	− 0.033	**0.434**	0.659
**Factor 3 Bothersome event (5 items) α = 0.69**				
10. I think men have advantage in not having menstruation	3.0 (1.3)	− 0.434	0.077	**0.520**
2. I think I expect extra consideration from people around me during menstruation	4.0 (1.1)	− 0.255	0.415	**0.449**
3. I feel that changes in physical condition during menstruation are greater than changes in normal physical condition	4.0 (1.1)	− 0.255	0.415	**0.449**
8. I think menstruation is something I just have to put up with	4.0 (0.8)	− 0.195	− 0.089	**0.448**
11. I think I hope it will be possible to get a short menstrual period	4.0 (1.2)	− 0.401	0.348	**0.406**

Factor loadings greater than 0.40 are in bold; protocol 4th edition

*M* mean, *SD* standard deviation

**Fig. 1:**
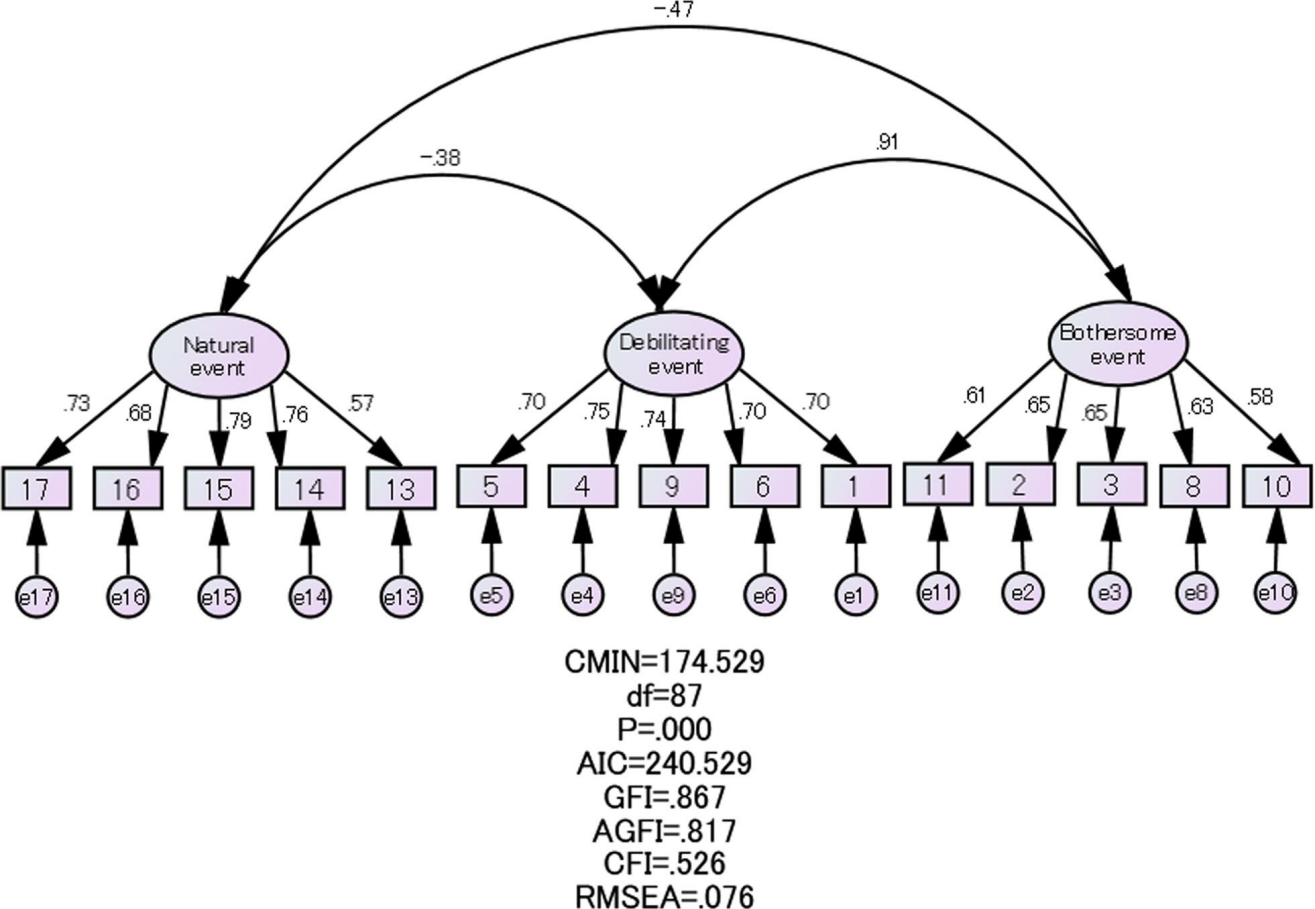
Confirmatory factor analysis of the 4th edition protocol (N = 176, Group 2). The observation variable ‘17, 16, 15, 14, 13 (Question 5 items)’ is affected by the latent variable ‘Factor 1 Natural event’. The observation variable ‘5, 4, 9, 6, 1 (Question 5 items)’ is affected by the latent variable ‘Factor 2 debilitating event’. The observation variable ‘11, 2, 3, 8, 10 (Question 5 items)’ is affected by the latent variable ‘Factor 3 Bothersome event’. Each observed variable is also affected by the error ‘e’. *CMIN/df* Chi-square/degrees of freedom, *AIC* Akaike’s Information Criterion, *GFI* goodness-of-fit index, *AGFI* adjusted goodness-of-fit index, *CFI* comparative fit index, *RMSEA* root mean square error of approximation

The background characteristics of Group 2, which were used in a confirmatory analysis, are presented in the right column of Table 2. The value indices were CMIN/df = 174.529/87 (P < 0.001), AIC = 240.529, GFI = 0.867, AGF = 0.817, CFI = 0.526, and RMSEA = 0.076 (95% CI = 0.59–0.92) (Fig. 1). Negative menstrual attitudes exhibited a 3-factor structure. A strongly positive correlation was observed between ‘Debilitating event’ and ‘Bothersome event’ (r = 0.91), whereas weakly negative associations were noted between ‘Natural event’ and ‘Debilitating event’ (r = − 0.38) and between ‘Natural event’ and ‘Bothersome event’ (r = − 0.47)’.

We also examined the difference in scores on the 15-item scale between performers and non-performers of each traditional menstrual practice (Table 4). For the three traditional menstrual practices—staying in their own house, going to public spaces, and general contact with others—scores on the 15-item scale were significantly higher in performers than in non-performers.

**Table 4:** Median comparison with negative menstrual attitudes and traditional menstrual practices (n = 176 in Group 2)

Traditional menstrual practices^a^ (n)	Total score	P^a^		Total score	P^a^		Total score	P^a^
	M (IQR)			M (IQR)			M (IQR)	
**Restriction of living places**								
Stay in own house			Go to public spaces			Stay in other’s house		
No (n = 159)	41.0 (8.0)	0.030	No (n = 31)	41.0 (8.0)	< 0.001	No (n = 75)	41.0(8.0)	0.462
Yes (n = 17)	45.0 (10.0)		Yes (n = 145)	46.0 (10.0)		Yes (n = 101)	42.0 (8.0)	
Use a bedroom			Use a kitchen					
No (n = 138)	41.5 (7.0)	0.388	No (n = 68)	42.0 (9.0)	0.631			
Yes (n = 38)	41.5 (10.0)		Yes (n = 108)	41.0 (8.0)				
**Restriction of contact with others**								
Contact with others generally			Contact with male family			Attend to male family		
No (n = 147)	41.0 (8.0)	0.006	No (n = 101)	42.0 (9.0)	0.304	No (n = 88)	42.0 (10.0)	0.613
Yes (n = 29)	45.0 (9.0)		Yes (n = 75)	41.0 (8.0)		Yes (n = 88)	41.0 (7.0)	
**Restriction of religious behaviors**								
Go to a temple			Join religious events			Attend a wedding ceremony		
No (n = 9)	38.0 (12.0)	0.165	No (n = 7)	38.0 (15.0)	0.404	No (n = 85)	42.0 (8.0)	0.122
Yes (n = 167)	42.0 (8.0)		Yes (n = 169)	42.0 (8.0)		Yes (n = 91)	41.0 (10.0)	

*M* median, *IQR* interquartile range

^a^Mann–Whitney U test

Table 5 shows the correlations between total scores on the 15-item scale and intensity of menstrual pain, indicating a positive correlation (r = 0.558). Moderately positive correlations were observed for ‘Debilitating event’ (r = 0.534) and ‘Bothersome event’ (r = 0.515), whereas a weakly negative correlation was noted for ‘Natural event’ (r = -0.187).

**Table 5:** Correlation between scores of the 4th edition protocol and menstrual pain intensity (n = 176, Group 2)

	Menstrual attitudes							
	Total Score	P^a^	Natural event*	P^a^	Debilitating event	P^a^	Bothersome event	P^a^
Intensity of menstrual pain	0.558	< 0.001	− 0.187	0.013	0.534	< 0.001	0.515	< 0.001

*Reversed item

^a^Spearman’s rank correlation coefficient

## Discussion

In this study, we developed a 15-item, 3-factor scale to evaluate negative menstrual attitudes among Nepalese women using exploratory factor analysis in consideration of their cultural and social background. The reliability and internal validity of this scale were adequate, but criterion reliability could not be examined due to the absence of a gold standard. Therefore, we preliminarily examined the relationship between negative menstrual attitudes evaluated using the 15-item scale and two parameters—traditional menstrual practices and menstrual pain—which are related to reproductive health.

The scale was deemed to accurately reflect the values of Nepalese women. For example, the item of ‘I can’t feel good during menstruation’ changed from ‘Bothersome event’ in the 4th edition protocol to ‘Debilitating event’ in the 15-item scale. Bothersome events predominantly reflect mental perceptions, whereas debilitating events reflect physical perceptions, suggesting that menstruation may be considered more of a physical difficulty than a mental difficulty in the Nepalese society. Indeed, approximately 70% of the participants reported menstrual pain. In contrast, the item ‘I think I expect extra consideration from people around me during menstruation’ changed from ‘Debilitating event’ in the 4th edition protocol to ‘Bothersome event’ in the 15-item scale. Nepalese women who perform traditional menstrual practices need to make special considerations for their surroundings due to behavioural restrictions [24] and may, therefore, consider this mentally bothersome.

Although the MAQ is the most widely used standard measure, menstrual attitudes differ between cultures [5]. In particular, an Indian version of the MAQ [7] was developed for Indian women who are culturally close to Nepalese; however, the 5-factor structure of the original MAQ did not hold. Therefore, it was necessary to develop a culturally appropriate scale for Nepalese women. The scale developed in this study was designed to be easily understandable with fewer questions and had a 3-factor structure based on the 5-factor structure of the original MAQ, allowing the new questionnaire to be answered by Nepalese women with low literacy levels rooted in the Nepalese culture. A qualitative study using the MAQ questions has also been conducted in Nepal [18], but even though the research subjects were highly educated women, they had difficulty understanding the content and answering the questions accurately. Thus, we believe that the present results indicate the usefulness of this newly developed scale to measure the negative menstrual attitudes of Nepalese women in consideration of their values.

In this study, 50.8% of participants had a low education level (< high school) and were able to answer the 4th edition protocol. Moreover, 40.9% of participants belonged to the higher castes (Brahmin and Chhetri), which was higher than the corresponding percentage in Nepal (28.8%) [25]. Reports have indicated that Nepalese women in the higher castes perform traditional practices more frequently than those in the lower castes [24, 26]. As such, we were able to conduct the study considering the literacy of Nepalese women who performed traditional menstrual practices.

As described above, the 15-item scale had several advantages for Nepalese women over other scales. First, the 15-item scale comprised fewer items than the original MAQ [3] (15 vs. 35 items) and was easy to answer. Second, the new scale considered the cultural background and literacy of Nepalese women and was easy to understand. Third, it could be applied in reproductive-aged women, whereas the original MAQ was only applicable in university students, and AMAQ [12] was only applicable in adolescent women.

Moreover, we conducted a confirmatory factor analysis to examine the structure of negative menstrual attitudes among Nepalese women. The P-value of CMIN was < 0.05, leading to the rejection of the null hypothesis. Although the CMIN did not exhibit a good fit, this result was dependent on the sample size. Versions of the MAQ in other languages have also reported CMIN with a P-value < 0.05 [2, 11]. The GFI (0.867) and AGFI (0.817) were also reported to be close to 0.9, of which the GFI was higher than the AGFI. The CFI was 0.526, which was < 0.9, whereas that of the British model was 0.52 [7, 11]. Additionally, the RMSEA was 0.076, which was < 0.1. We, therefore, considered that the model goodness-of-fit indices in this study were within permissible ranges. Confirmatory factor analysis also revealed the 3-factor (‘Debilitating event’, ‘Bothersome event’, and ‘Natural event’) structure of negative menstrual attitudes among Nepalese women. We observed a positive correlation between ‘Debilitating event’ and ‘Bothersome event’, whereas ‘Natural event’ was negatively correlated with other factors. In this regard, ‘Natural event’ may be an independent factor to the other two factors.

We examined traditional menstrual practices and menstrual pain, which have been associated with negative menstrual attitudes, as these parameters are reflective of reproductive health issues among Nepalese women. Scores for three traditional menstrual practices (‘stay in own house’, ‘go to public spaces’, and ‘contact with others generally’) were higher in performers than in non-performers, suggesting that negative menstrual attitudes evaluated using the 15-item scale highlighted behavioural targets that can reduce the frequency of traditional menstrual practices adversely affecting reproductive health among Nepalese women. In contrast, the frequency of traditional menstrual practices related to religious behaviour was not associated with negative menstrual attitudes. Most Nepalese women believe that they should obey restrictions on religious behaviour and avoid places of worship or religious events during menstruation [24]. Therefore, these results indicated that traditional menstrual practices related to religious behaviour were not associated with negative menstrual attitudes among Nepalese women.

Furthermore, we observed a significantly positive correlation between the total scores of the 15-item scale and the intensity of menstrual pain, which was consistent with the findings of previous reports [3–5]. Among the three subscales, moderately positive correlations with intensity of menstrual pain were observed in the scores of ‘Debilitating event’ and ‘Bothersome event’, whereas a weakly negative correlation was observed for the scores of ‘Natural event’, suggesting that education to improve negative menstrual attitudes may contribute to a reduction in perceived menstrual pain. In this regard, comprehensive evaluation of negative menstrual attitudes among women is essential to promote women's rights to social participation and reproductive health.

## Limitations

Despite these findings, this study had some limitations. The first is a limitation regarding low literacy. In this study, we developed a scale that considered Nepalese culture and low literacy among Nepalese women. However, they needed the help of a research collaborator to understand the scale accurately, which could have caused bias in their responses. In the future, it is necessary to make the scale even easier to understand for Nepalese women and to standardise the assistance methods for research collaborators. The second is a limitation regarding the generalisation of the results. The criterion validity was not examined because no appropriate criterion scale was available. Additionally, we enrolled participants via snowball sampling, and recruitment was performed through community meetings such as the Lions Club meetings in Pokhara (metropolitan area) and mother-group meetings in Damauli (municipal area) during the study period. This resulted in recruitment of an unequal number of participants from two distinct populations; therefore, subject enrolment could be considered biased in this study. In the future, it is necessary to examine the generalizability of the results in a large-scale survey using this scale. Moreover, these large-scale surveys must also consider the differences in the structure of menstrual attitudes based on castes and place of residence. Furthermore, although the scale was developed for women of reproductive age, its applicability in adolescent women is another issue that needs to be addressed in further studies.

## Conclusions

This study is the first to evaluate menstrual attitudes and analyse the factorial structure of these attitudes among Nepalese women. The main outcomes of the study were as follows: (1) we developed a 15-item scale to evaluate negative menstrual attitudes among Nepalese women, (2) negative menstrual attitudes among Nepalese women had a 3-factor structure, (3) correlations were observed between the negative menstrual attitudes evaluated using the 15-item scale and the frequencies of certain traditional menstrual practices, as well as intensity of menstrual pain. Our study indicates that the evaluation of negative menstrual attitudes in women may be a key factor in promoting women's rights for social participation and reproductive health. Our analysis also reveals that the 15-item scale may be useful for improving reproductive health among Nepalese women. To more comprehensively examine the relationship between negative menstrual attitudes and reproductive health issues in the future, it will be necessary to conduct large-scale surveys in Nepal using the same 15-item scale.

## Data Availability

The datasets used in this study are available with the corresponding author on reasonable request.

## Abbreviations

AGFI: Adjusted goodness-of-fit index
AIC: Akaike’s information criterion
AMAQ: Adolescent Menstrual Attitude Questionnaire
CFI: Comparative fit index
CMIN: Chi-square value
GFI: Goodness-of-fit index
JICA: Japan International Cooperation Agency
KMO: Kaiser–Meyer–Olkin
MAQ: Menstrual Attitudes Questionnaire
RMSEA: Root mean square error of approximation
